# Comprehensive Assessment of Physiological and Psychological Responses to Virtual Reality Experiences

**DOI:** 10.1089/jmxr.2024.0020

**Published:** 2024-10-31

**Authors:** Valentin Fauveau, Anastasia K. Filimonov, Renata Pyzik, James Murrough, Laurie Keefer, Omer Liran, Brennan Spiegel, Filip K. Swirski, Zahi A. Fayad, Wolfram C. Poller

**Affiliations:** 1 Biomedical Engineering and Imaging Institute (BMEII), Icahn School of Medicine at Mount Sinai, New York, New York, USA.; 2 Cardiovascular Research Institute, Icahn School of Medicine at Mount Sinai, New York, New York, USA.; 3 Department of Psychiatry, Depression and Anxiety Center for Discovery and Treatment, Icahn School of Medicine at Mount Sinai, New York, New York, USA.; 4 Mount Sinai School of Medicine, Friedman Brain Institute, New York, New York, USA.; 5 Department of Neuroscience, Icahn School of Medicine at Mount Sinai, New York, New York, USA.; 6 VISN 2 Mental Illness Research, Education, and Clinical Center (MIRECC), James J. Peters VA Medical Center, Bronx, New York, USA.; 7 The Henry D. Janowitz Division of Gastroenterology, Icahn School of Medicine, New York, NY, USA.; 8 Psychiatry, Cedars-Sinai Medical Center, Los Angeles, California, USA.; 9 Health Services Research, Cedars-Sinai Medical Center, Los Angeles, California, USA.; 10 Cardiology Division and Cardiovascular Research Center, Massachusetts General Hospital, Harvard Medical School, Boston, Massachusetts, USA.

**Keywords:** virtual reality, medicine, stress, physiological signals, immune system, psychological tests

## Abstract

Virtual reality (VR) has emerged as a technology with huge improvements in the last few years, offering immersive experiences and paving the way for novel applications across various fields. In the medical field, VR is advancing at a notable rate, allowing for new innovations ranging from pain treatment to rehabilitation and showing its potential to reconfigure the brain to modify how our mind perceives our body and environment. The immersive nature of VR can be reflected in people’s physiological and psychological responses, making stress management a key focus to obtain potentially transformative effects as a regulatory stress treatment. This pilot study focuses on a comprehensive assessment, both physiologically and psychologically, of the impact of stressful versus relaxing VR experiences. Our exploratory study examines the effectiveness of this emerging technology in manipulating stress levels in a group of 20 healthy volunteers. In a randomized cross-over design, each subject was exposed to both experiences in two independent sessions, during which their physiological signals were continuously measured. Additionally, blood draws and psychological tests were obtained before and after each VR experience. The results show physiological changes consistent with the paradigm of the experience and supported by self-reported psychological scores. Laboratory findings reveal statistical differences in cortisol levels when comparing changes in the Stress versus Relax experience. Additionally, statistically significant changes in white blood cell counts are observed when comparing pre- versus post-Stress VR. These results provide a first attempt to explore how VR scenarios can modulate individual stress levels and the immune response. Future exploratory avenues may include the implementation of VR-based treatments for stress modulation aimed at mitigating the detrimental effects of stress on mental health, cardiovascular disease, and the immune system.

## Introduction 

Virtual reality (VR) stands as an emerging immersive technology using computer-generated simulations that engage users in virtual worlds. While the current immersive experience primarily involves vision and hearing, ongoing development efforts aim at incorporating additional senses to continually enhance the technology, achieving even more realistic experiences.^1^ By the manipulation of the human senses, VR generates dynamic scenarios triggering physiological and psychological responses.^2^

VR has progressively solidified its role in advancing medicine, demonstrating promising results across various disciplines. These include medical training, where studies have shown its higher efficacy in training personnel for clinical decision-making^3^ and instructing new medical students in orthopedic surgery.^4^ Cognitive and motor disease rehabilitation, offering accessibility, comfort (home settings), and promising outcomes.^5^ Medical diagnosis and treatment of mental disorders,^6^ with convincing evidence in anxiety disorders and posttraumatic stress disorders. The use of VR in stress management is emerging as a promising and engaging tool,^7^ showing its potential to be used as a stress regulator.

Previous studies have investigated the effects of different virtual mediums on producing emotions and influencing stress levels. Notably, Dr. Giuseppe Riva and his group explored the impact of VR on human emotions, demonstrating how imagery techniques through VR can evoke emotions and a sense of presence in participants.^8^ Presence is defined as the sensation of being fully embedded in a virtual world, where the limit between real and virtual disappears. In their study, they presented three VR scenarios (anxious, relaxing, and neutral) to a group of 61 students, measuring their anxiety and presence levels. Their findings indicate that VR is an effective medium for mood induction, capable of eliciting specific emotional states aligned with the VR content.

More recent studies have analyzed the differences in physiological and psychological responses across different virtual mediums.^9,10^ The study by Vatsal R. and Mishra S. examined the physiological and psychological responses between VR and flat screen (FS) games,^9^ finding that VR games induce stronger emotions, higher arousal, increased cognitive load, and stress, but lower dominance compared to FS games. The research available on the physiological and psychological effects of VR games supports the idea that VR technology may serve as an effective medium for stress management.

Stress has a significant impact on both health and disease.^11,12^ It represents an emotional response generated in the brain, integrating various external and internal inputs. The brain receives information about the external environment through sensory organs, while internal sensors report physiological parameters. Based on the available data and adaptive strategies, the brain triggers a stress response transmitted to the periphery via its output systems.^13–15^ The two primary stress systems are the hypothalamus–pituitary–adrenal axis, which regulates cortisol release from the adrenal gland cortex, and the sympathetic nervous system (SNS), responsible for innervating peripheral organs and releasing noradrenaline locally from nerve endings and systemically from the adrenal gland medulla. Both systems exert profound effects on the immune system, extensively documented in the literature.^13,14^

There are various stress categories based on duration, source, and response.^15^ Chronic stress occurs when a stressor persists over a long period of time, and it is widely acknowledged to have adverse effects on the immune system and its association with various diseases.^16^ In contrast, acute stress is a short-term stress obtained from an event or a situation, triggering physiological responses such as increased heart rate (HR) and adrenaline release. Some evidence suggests that episodes of acute stress may enhance the immune system’s ability to combat invading bacteria^17,18^ and generate antibodies against pathogens.^19^

Numerous studies have highlighted the role of stress in modulating the immune response, impacting an individual’s susceptibilities to external pathogens. VR emerges as a promising non-invasive tool for managing stress levels.^20^ By immersing subjects in vivid scenarios that can elevate or alleviate stress, VR offers the potential to alleviate chronic stress through relaxing VR experiences. Alternatively, it can also be utilized to intentionally induce acute stress through stressful VR games, aiming at potentially improving the immune system’s response.

This pilot study aims at conducting a comprehensive physiological and psychological evaluation of VR experiences designed to induce either stress or relaxation. The evaluation includes continuous physiological measurements during the experiences, as well as psychological tests (the State-Trait Anxiety Inventory [STAI] and the Anxiety Sensitivity Index-3 [ASI-3]) and blood collections before and after each experience to analyze cortisol levels and leukocyte count. The study explores the following hypotheses: (1) The relaxing VR experience alleviates subjective stress levels, reduces changes in physiological parameters, lowers self-reported psychological test scores, also affecting the immune system by decreasing plasma cortisol, and increasing blood leukocyte levels in healthy subjects. (2) The stressful VR experience induces acute stress, leading to an increase in physiological parameters and self-reported psychological test scores, thereby affecting the immune system by increasing plasma cortisol and decreasing blood leukocyte levels in healthy subjects. The study received approval from the Institutional Review Board at Mount Sinai Hospital in New York City. All participants were given an overview of the study, and written consent was obtained prior to the start. To ensure the privacy of the participants, all data was anonymized.

## Materials and Methods

### Study devices

#### VR headset: Meta Quest 2

**FIG. 1.**
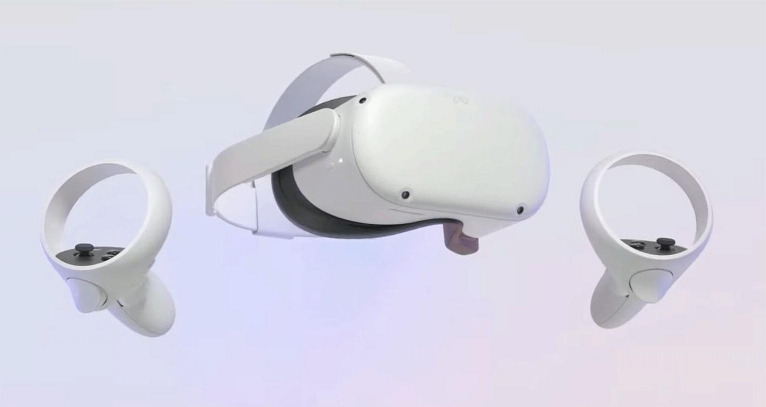
Meta Quest 2 (Oculus) VR headset and touch controllers. *Image obtained from:* https://www.amazon.com/-/es/Meta-Quest-All-One-Oculus-2/dp/B099VMT8VZ?th=1 *(accessed on March 2024)*. VR, virtual reality.

In this research, we employed the commercially available VR headset and Meta Quest 2 (Oculus) (see Fig. 1). This VR headset tracks the position of the user’s head and hands, enabling free movement, and interaction within the digital space. The realistic physics and high resolutions combine to create immersive worlds, providing users with distinctive and engaging experiences.

#### Physiology measurement: Biopac system

**FIG. 2.**
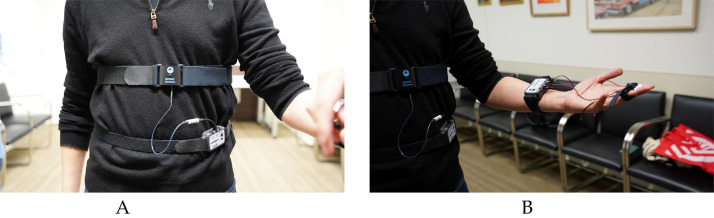
Biopac System set up. **(A)** Electrocardiogram (ECG) and respiration (RSP) transmitter. **(B)** Electrodermal activity (EDA) and photoplethysmography (PPG) transmitter.

To record physiological signals, we used the Biopac System Inc., a data acquisition system designed for life science research. This system facilitates the simultaneous and continuous recording of multiple physiological signals, such as electrocardiogram (ECG), respiration (RSP), electrodermal activity (EDA), and photoplethysmography (PPG). The system includes one receptor connected to the computer, two transmitters (one for RSP and ECG, see Fig. 2A; the other for EDA and PPG, see Fig. 2B), one RSP transducer and electrodes.

### VR games

Our goal was to find both a stress-inducing and a relaxing VR experience, each suitable for approximately 30 min of use, with straightforward gameplay (almost no use of controllers) that requires minimal onboarding instructions. Based on these criteria, the final candidate for the Stress VR experience was “Affected: The Manor Complete Edition,” a haunted house exploration game. For Relax VR we selected “TRIPP,” a ∼30-min meditation VR game.

#### Stress VR experience

**FIG. 3.**
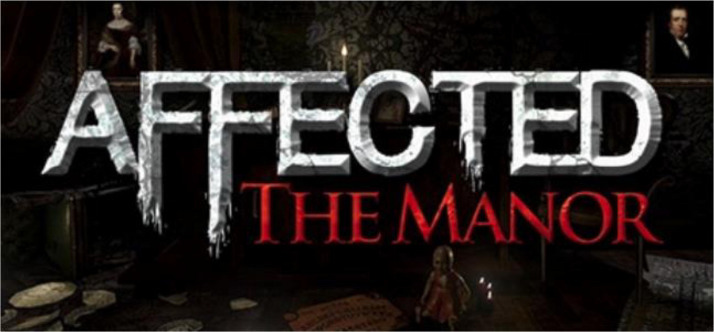
Stress VR Game: “Affected: The Manor Complete Edition.” Available on Meta Quest 2 and Steam. *Image obtained from:* https://store.steampowered.com/app/707580/AFFECTED_The_Manor__The_Complete_Edition/ *(accessed on March 2024)*. VR, virtual reality.

For the stressful VR experience, we opted for the ∼30-min VR game titled “Affected: The Manor Complete Edition” (see Fig. 3). This VR horror experience ventures users through a haunted house featuring various spooky situations and jump scares. The gameplay involves a basic walkthrough, using a flashlight, and opening doors. For a more realistic experience, participants engaged in the game while standing up, allowing them to move freely in space. This approach aimed at creating a more immersive experience, simulating the feeling of being inside the haunted house.

#### Relax VR experience

**FIG. 4.**
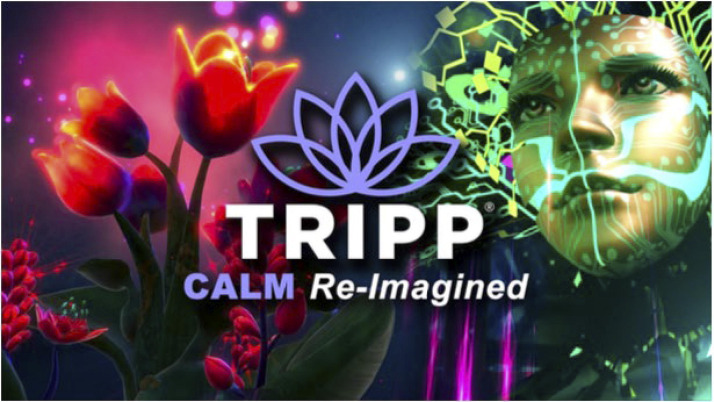
Relax VR Game: “TRIPP.” Available on Meta Quest 2 and Steam. *Image obtained from:* https://www.tripp.com/ *(accessed on March 2024)*. VR, virtual reality.

The relaxing VR experience involved an immersive meditation session featuring calm music and peaceful environments. The subjects were exposed to a ∼30-min VR relaxation game called “TRIPP” (see Fig. 4). Throughout these sessions, participants remained seated, contemplating calm surroundings and engaging in breathing exercises. Previous studies have demonstrated the effectiveness of this game in evoking calmness and relaxation.^21^

### Subject population

This pilot study, employing a longitudinal, open-label, and cross-over design, involved the prospective recruitment of healthy subjects to participate in VR experiences. A total of 22 participants were recruited from Mount Sinai Facilities, including students, volunteers, and employees. Two subjects withdrew from the study due to dizziness and uneasiness during the stress VR experience, resulting in a final cohort of 20 individuals (8 males, 12 females, mean age = 30 ± 4.75 years). The cohort also exhibited diversity: 40% White, 30% Asian, 10% African American, and 20% Other (Table 1).

**Table 1.** Demographics

Individual variables	*N*
Gender	
Male	8
Female	12
Race	
White	8
African American	2
Asian	6
Other	4
Age	30 ± 4.75 years

The study’s inclusion criteria specified subjects aged 18 years old and above who could read and sign the informed consent. The exclusion criteria encompassed individuals who were unable to communicate/read in English, unable to wear, or independently maneuver the VR headset, subjects diagnosed with vertigo, migraines, seizures or self-reported severe sensitivity to motion, severe vision or hearing impairments, underlying chronic medical conditions, or subjects taking medications affecting physiological functions.

Recruitment was conducted by posting flyers at the Mount Sinai facilities and by word of mouth. The team then contacted interested volunteers to explain the study details. If a participant met the inclusion criteria, a convenient time was arranged for a detailed explanation and signing of the consent form. All questions were carefully answered to ensure that participants understood the study’s purpose, potential risks, and benefits before giving consent.

The risks involved a brief period of psychological stress/distress, in line with the study’s objectives. Participants were informed that they could remove the VR headset at any point to terminate their participation. Additionally, two investigators were present with the participant throughout the experience to manage any potential risks. In case of dizziness, we assisted the participants in removing the headset, seated them, and provided water until they recovered. Potential privacy risks related to information disclosure were also detailed. Lastly, the risks associated with blood draws were explained, including pain, bruising, and a slight risk of infection.

Participants were compensated with a $25 gift card for each study visit they successfully completed.

### Study design

**FIG. 5.**
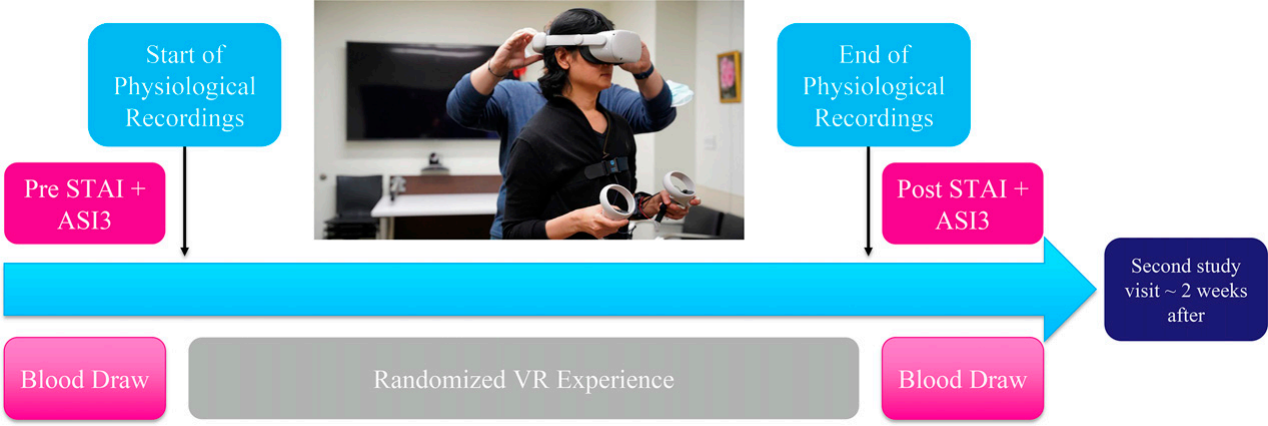
Study design protocol. The study was conducted over a period of approximately 2 weeks. During each visit, we began by drawing blood through peripheral venipuncture. Next, participants completed the psychological tests, followed by measuring their skin temperature and locating the wearables to collect physiological data. We then set up the experience, placed the VR headset on the participant and started the physiological recordings. After the session, we removed the VR headset and wearables, took the temperature, provided again the psychological tests and took a second blood sample. VR, virtual reality.

The study was performed over a period of approximately 2 weeks per participant (14 ± 4.5 days) between June and July 2023. Participants were randomly assigned to either the stress or relax VR experience during their first visit, followed by the respective opposite experience during their second visit. The randomization was done by flipping a coin at the first visit, at that moment the subjects were informed about which VR experience they would undergo first. Blood draws and psychological self-reported stress levels (ASI-3 and STAI) were measured during each visit before and after the VR experience, while physiological parameters (ECG, RSP, PPG, and EDA) were recorded continuously during the session (Fig. 5).

### Physiological data analysis

Four different physiological signals were recorded during the VR experiences using the Biopac System Inc. These correspond to ECG, PPG, RSP, and EDA. ECG and PPG were utilized to analyze HR, RSP enabled the examination of respiratory rate (RR), and EDA offered insights into sweat variability by measuring changes in the skin’s electrical conductance. We also discreetly measured skin temperature before and after each experience.

To analyze HR, we opted to use the ECG signal exclusively, as the PPG data produced significant noise due to the continuous pressure on the sensor by the VR controls. While the ECG signal provides a graphic representation of cardiac activity, the presence of noise can significantly hinder visual diagnosis and feature extraction. Several post-processing methods have been proposed in the literature to denoise and extract the efficient morphology of ECG signals.^22–24^ For our analysis, we applied a wavelet thresholding technique with the Daubechies 4 wavelet function and a decomposition level 4. After denoising the signal, we used the “ecg_process” function from the Python neurokit2 module^25^ to extract the HR.

RSP is a slow frequency signal specifically designed to measure RSP activity associated with changes in thoracic wall motion. During inspiration, the sensor stretches, increasing the output level, while expiration causes the sensor to relax leading to a drop in the output signal level. To calculate and analyze the RR, it is necessary to denoise the RSP to correct for motion artifacts. Given the immersive nature of VR experiences, motion is an artifact inherently associated with this signal. Therefore, to extract the RR, we applied a low-pass finite impulse response moving average filter. Given that the normal RSP rate is a slow signal between 6 and 20 breaths/min, we set the threshold of the low pass filter to 1 Hz. Subsequently, we extracted the RR using the neurokit2^25^ and the “rsp_process” function.

EDA is the measurement of skin conductance, which is influenced by sweat secretion of the eccrine sweat glands. This process is modulated by the SNS, triggered by environmental stimuli involving various senses and producing an involuntary response from sweat glands in the hypodermis. Sweat is a well-known indicator of stress levels,^26^ its increase leads to a direct elevation in the skin’s electrical conductance. The EDA signal is mainly composed of two components: the general tonic level, which relates to slow changes in skin conductance over time indicative of general changes in autonomic arousal, and the phasic component, representing faster changes in the signal related to specific external stimuli.^27^ For EDA’s signal analysis, it is important to acknowledge that this signal shows a different baseline that is individual-dependent. Therefore, some researchers have concluded that the skin conductance baseline level, on its own, may not be highly informative.^28,29^ To address the high variability in EDA values, we normalized the data using the *z*-score. From the normalized data, we analyzed the net integral sum, offering insights into the net change over time. Additionally, we calculated the slope of the normalized EDA signal to gain an understanding of the overall trend.

### Lab tests analysis

Before and after each VR session, blood samples were collected from the subjects using peripheral venipuncture. We conducted analyses of cortisol levels and white blood cell (WBC) count pre- and post-VR exposure to evaluate changes induced by the VR experience. These measurements provided insights into the potential use of VR experiences as mood-regulating tools to intentionally influence not only cortisol levels but also leukocyte quantity.

### Psychological data analysis

Stress perception was another measure analyzed in the study. To explore how subjects perceived both VR experiences, we provided two different psychological examinations before and after the sessions. These included the STAI and ASI-3.

The STAI^30,31^ is a widely used test measuring state (Y-1) and trait (Y-2) anxiety levels. The range of possible scores varies from a minimum score of 20 to a maximum score of 80 on both subscales. Scores are commonly classified as “low anxiety” (score: 20–37), “moderate anxiety” (score: 38–44), and “high anxiety” (score: 45–80). STAI Y-1 (state anxiety) is a sub-scale that evaluates a person’s current or temporary anxiety level, reflecting changes in response to specific situations or circumstances. It aims to measure how an individual feels at a particular moment in time, making it useful for assessing the intensity of anxiety in response to a specific event or stressor. STAI Y-2 (trait anxiety) is a sub-scale measuring an individual’s general or stable tendency to experience anxiety over time. It assesses how prone someone is to experiencing anxiety in various situations, regardless of the specific circumstances. The STAI Y-2 is designed to measure the overall propensity for anxiety as a personality trait.

The ASI-3^32^ is a self-report assessment of the anxiety sensitivity reflecting one’s tendency to misinterpret the meaning of anxiety-relevant sensations. The test corresponds to an 18-item self-report questionnaire composed of three subscales: physical, cognitive, and social. The total scores range from 0 to 72, the higher the value, the higher the anxiety sensibility. And by analyzing the sub-scale results we can identify the subscale which generates the greater concern for subjects.

### Statistical analysis

Given the small sample size and the data’s failure to meet the normality assumption, we opted for the non-parametric Wilcoxon signed-rank test to analyze both the physiological and psychological values. For the continuous physiological values, we compared the Relax versus Stress VR outcomes. For the parameters obtained before and after the sessions (skin temperature, lab results, and psychological tests), we conducted the Wilcoxon signed-rank test with repeated measures across the four time points: pre-Relax, post-Relax, pre-Stress, and post-Stress. To mitigate the risk of Type I error (false positive), we employed the Bonferroni–Holm correction method.

To validate all the statistically significant results (*p* value ≤ 0.05) we conducted a randomization test. This involved calculating the differences for paired observations and randomly changing the sign, thereby changing the order of observed changes. We performed 1000 permutations, running the Wilcoxon signed-rank test for each permutation. We then assessed the proportion of permuted statistics with a more extreme *p*-value than the observed one. This proportion represents the *p*-value of the randomization test, confirming that the statistically significant results were not obtained by chance.

Lastly, we conducted a correlation analysis to further explore the dynamics between the physiology and psychology parameters, aiming to uncover links specific to each VR experience. To account for changes within each subject pre- and post-experience, we calculated the delta (post−pre) for all the variables acquired before and after the session. Subsequently, we performed a pairwise correlation coefficient analysis for all variables within each VR experience. For this analysis, we employed the non-parametric Spearman correlation method, which measures the monotonic relationships.

## Results

### Physiological results

**FIG. 6.**
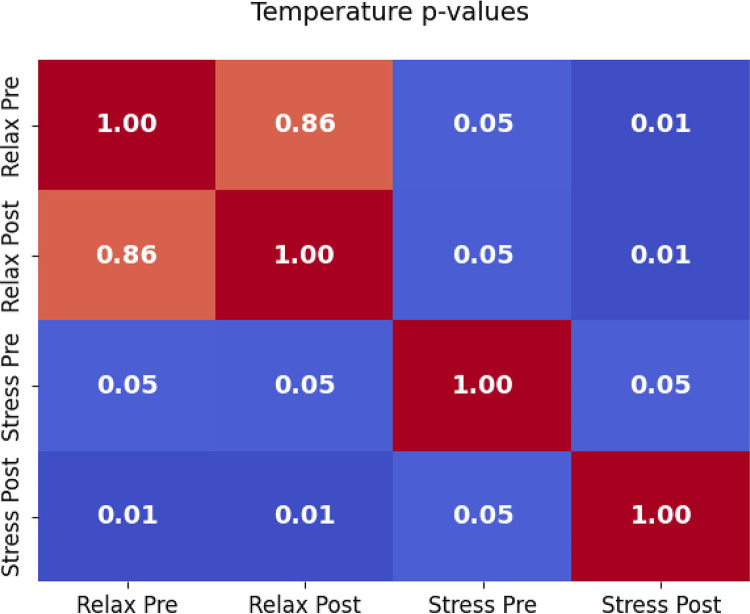
Statistical significance of temperature values. This figure shows the *p* values obtained from analyzing the skin temperature at the four time points. For the Relax VR experience, the mean temperature was 97.2 ± 1 pre-experience, and 97.2 ± 0.96 post-experience. For the Stress VR experience, the mean temperature was 98 ± 0.56 pre-experience and 98.3 ± 0.5 post-experience. VR, Virtual reality.

The results of skin temperature can be observed in Figure 6. There was a small yet significant increase post-experience in Stress VR, averaging 98.3 ± 0.5 F compared to 98 ± 0.56 F pre-session (*p*-value = 0.05). Conversely, there was no significant change in skin temperature for Relax VR, with a consistent average of 97.2 F before and after the experience (*p*-value = 0.86). When comparing the temperature pre-session between both experiences, a statistical difference was observed (*p*-value = 0.05), likely influenced by the psychological predisposition of subjects knowing the type of experience they were about to undergo.

**FIG. 7.**
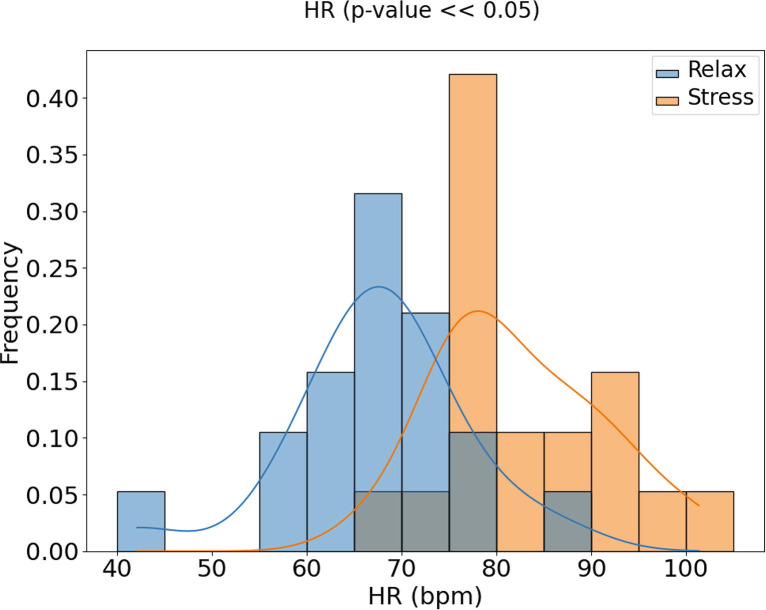
Average ECG HR distribution results per experience. The distributions display the observed frequency of the average HR obtained for the entire cohort. Among all participants, the average HR for Relax VR was 67.3 ± 9.0 bpm, while for Stress VR it was 82.3 ± 8.5 bpm. The *p*-value for comparing the heart rates between both experiences was 0.0001. ECG, electrocardiogram; HR, heart rate; VR, virtual reality.

The ECG HR, reflecting the average HR during the entire session was notably higher for Stress VR, with an average of 82.3 ± 8.5 bpm versus 67.3 ± 9.0 bpm for Relax VR (*p*-value = 0.0001) (Fig. 7).

**FIG. 8.**
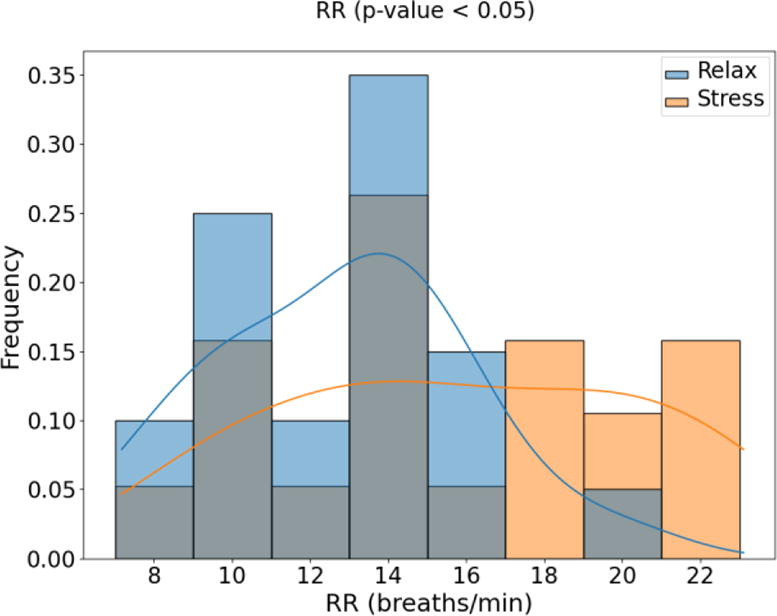
Average RSP respiratory rate (RR) distribution results per experience. The distributions depict the observed frequency of average RR obtained for the entire cohort. Among all participants, the average RR for Relax VR was 12.7 ± 3.1 breaths/min, while for Stress VR it was 15.9 ± 4.5 breaths/min. The *p*-value for comparing the RRs between both experiences was 0.01. VR, virtual reality.

The RR also displayed a significant difference between the two experiences. In Relax VR, the average RR was 12.7 ± 3.1 breaths/min, while in Stress VR it corresponded to 15.9 ± 4.5 breaths/min (*p*-value = 0.01) (Fig. 8).

**FIG. 9.**
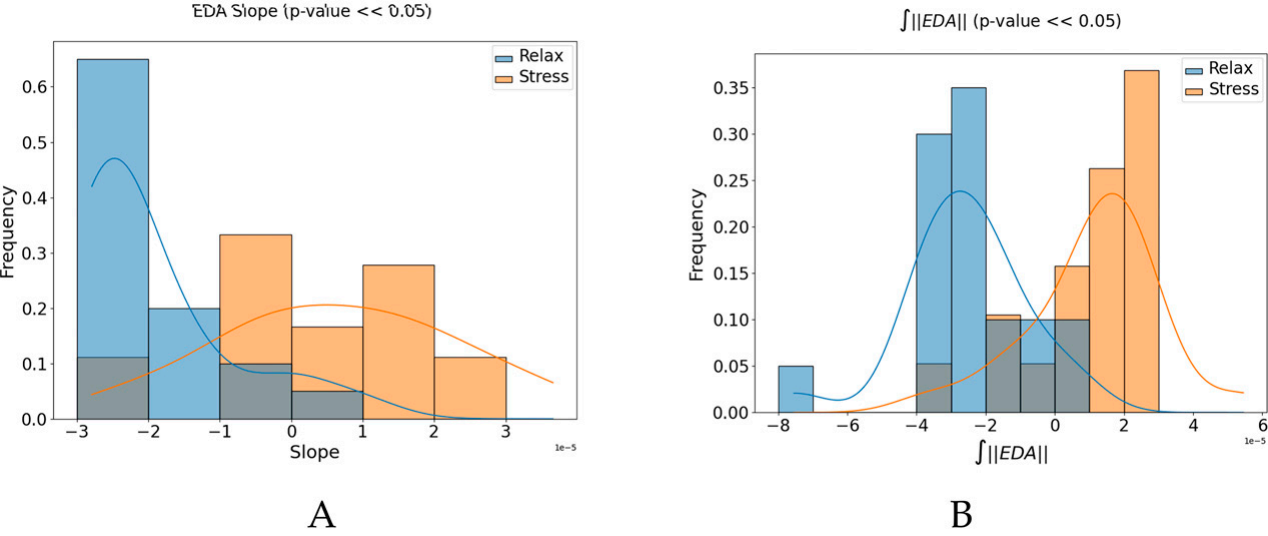
Analysis of the normalized electrodermal activity (EDA) signal. **(A)** Distributions of the observed slope of the normalized EDA signal (trend) for the entire cohort. For Relax virtual reality (VR), we observed on average a negative slope, showing a downward trajectory trend. For Stress VR, we observed on average a positive slope, indicating an upward trajectory trend. The *p*-value for comparing the EDA trend between both experiences was 0.0001. **(B)** Distributions of the integral of the normalized EDA signal (net change) for the entire cohort. For Relax VR we observed on average a negative net change, indicating a decrease in sweat secretion. For Stress VR we observed on average a positive net change, suggesting an increase in sweat secretion. The *p*-value for comparing the EDA net change between both experiences was 0.0001.

The EDA showed significant differences in sweat secretion. For Stress VR, there was a positive net change, indicating an increase in sweat secretion, while for Relax VR, the net change was negative, suggesting a decrease (*p*-value = 0.0001). This physiological effect was also reflected in the trend of the signal, showing an upward trajectory for Stress VR and a pronounced downward trend for Relax VR (*p*-value = 0.0001) (Fig. 9).

### Laboratory results

**FIG. 10.**
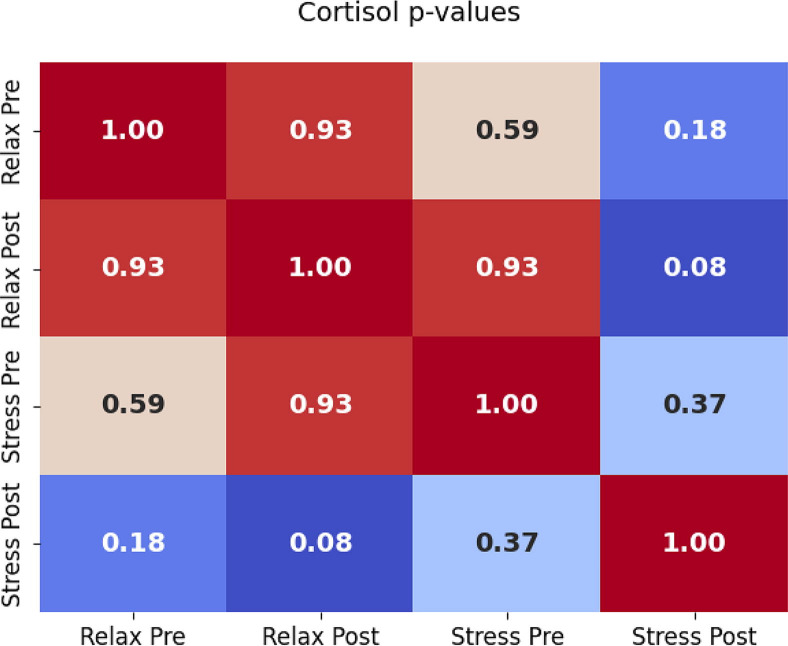
Statistical significance of cortisol levels. This figure presents the *p* values obtained from analyzing the Cortisol levels at the four time points. Cortisol levels during the Relax VR experience averaged 8.4 ± 2.85 mcg/dL pre-experience, and 9.2 ± 4.14 mcg/dL post-experience. Cortisol levels during the Stress VR experience averaged 9.55 ± 3.5 mcg/dL pre-experience, and 12.1 ± 5.34 mcg/dL post-experience. VR, virtual reality.

When comparing the four time points for the cortisol levels, no significant result was obtained (Fig. 10). The lowest *p*-value was achieved by comparing post-Relax to post-Stress, yet the differences were not statistically significant (*p*-value = 0.08). Consequently, an additional analysis was conducted to check for statistical differences in the delta (post − pre) between both experiences. This test revealed a *p*-value of 0.04, indicating a statistically significant change in cortisol levels from a baseline when participants were exposed to these two VR experiences. On average, cortisol levels increased by 0.8 mcg/dL in Relax VR and by 2.55 mcg/dL in the Stress VR experience.

**FIG. 11.**
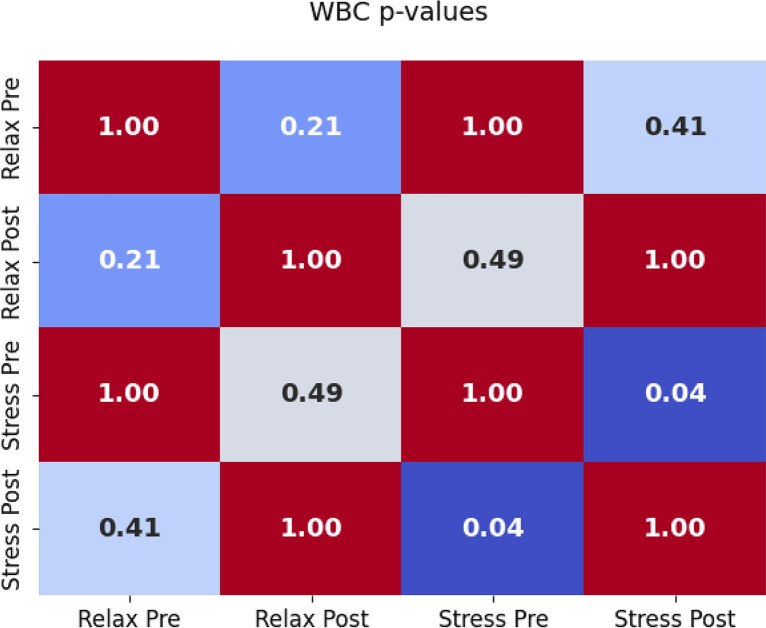
Statistical significance of WBC count. This figure depicts the *p* values obtained from analyzing the WBC count at the four time points. The WBC count for Relax VR averaged 6.6 ± 1.6 × 10^3^/μL pre-experience, and 7 ± 1.6 × 10^3^/μL post-experience. WBC count for Stress VR averaged 6.6 ± 1.4 × 10^3^/μL pre-experience, and 7 ± 1.5 × 10^3^/μL post-experience. VR, virtual reality; WBC, white blood cell.

The WBC count revealed in both experiences an average increase from 6.6 × 10^3^/μL pre-session to 7 × 10^3^/μL post-session. However, statistically significant changes were observed only when comparing pre-Stress versus post-Stress with a *p*-value of 0.04 (Fig. 11). When comparing pre-Relax versus post-Relax, the null hypothesis could not be rejected (*p*-value = 0.21), indicating no significant differences between the paired observations.

### Psychological results

The psychological examinations also unveiled statistically significant changes in perceived stress levels when comparing the four time points.

**FIG. 12.**
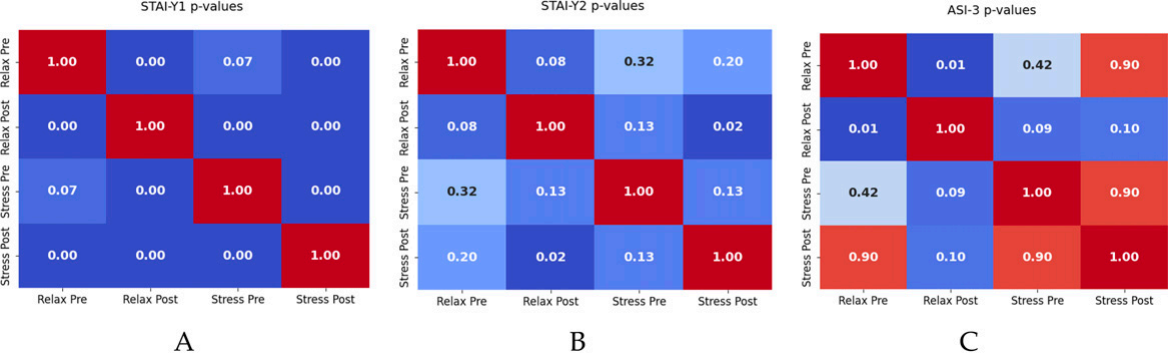
Statistical significance of psychological tests (STAI and ASI-3). **(A)** *p* values obtained from the statistical analysis of the STAI-Y1 values at the four time points. STAI-Y1 scores for Relax VR pre-experience = 28.65 ± 5.0 and post-experience = 25.3 ± 4.1. STAI-Y1 scores for Stress VR pre-experience = 30.65 ± 6.2 and post-experience = 40.45 ± 11.36. **(B)** *p* values obtained from the statistical analysis of the STAI-Y2 scores at the four time points. STAI-Y2 scores for Relax VR pre-experience = 36.85 ± 11.53 and post-experience = 33.25 ± 11.25. STAI-Y2 scores for Stress VR pre-experience = 35.5 ± 9.7 and post-experience = 38.2 ± 12.13. **(C)** *p* values obtained from the statistical analysis of the ASI-3 scores at the four time points. ASI-3 scores for Relax VR pre-experience = 13.25 ± 9.19 and post-experience = 9.0 ± 8.5. ASI-3 scores for Stress VR pre-experience = 11.85 ± 9.0 and post-experience = 12.9 ± 11.68. VR, virtual reality.

The STAI-Y1, measuring the person’s current or temporary anxiety levels, showed significant differences between all the paired observations except when comparing the results of pre-Relax versus pre-Stress (*p*-value = 0.07) (see Fig. 12A). During Relax VR, the STAI-Y1 results decreased from 28.65 ± 5.0 pre-experience to 25.3 ± 4.1 post-experience (*p*-value: 0.001). In contrast, it significantly increased during Stress VR, with an average score of 30.65 ± 6.2 pre-experience versus 40.45 ± 11.36 post-experience (*p*-value: 0.0004).

As for the STAI-Y2 test, measuring the individual’s general tendency to experience anxiety, we only observed a significant difference when comparing post-Relax to post-Stress with a *p*-value of 0.02. During Relax VR, the STAI-Y2 scores decreased from 36.85 ± 11.53 pre-experience to 33.25 ± 11.25 post-experience (*p*-value: 0.08). For Stress VR, the scores increased on average from 35.5 ± 9.7 to 38.2 ± 12.1 (*p*-value: 0.13) (see Fig. 12B). Given that the only significant statistics for this measurement were obtained when comparing the post-experience values (Post-Stress vs. Post-Relax), we decided to conduct an additional statistical test to measure the delta (post − pre) and compare it between both experiences. This statistical test revealed a statistically significant difference, with a *p*-value of 0.007. On average, the values decreased by 3.6 points in the Relax experience, while they increased by 2.7 points in the Stress VR experience.

The final psychological assessment, ASI-3, indicating the person’s primary source of anxiety, yielded only significant results for Relax VR but not for Stress VR. While the psychological scores significantly decreased on average from 13.25 ± 9.19 pre-experience to 9.0 ± 8.5 post-experience for Relax VR (*p*-value: 0.01); for Stress VR, we observed an average increase from 11.85 ± 9.0 to 12.9 ± 11.68 but the results were not statistically significant (*p*-value: 0.9) (see Fig. 12C).

### Randomization test

All the statistically significant results were confirmed by performing a randomization test to validate that these results were not obtained by chance (see Table 2).

**Table 2.** Randomization test results

Randomized variable	Randomization test
	*p* value
Physiological signals	
Temp (Relax Pre vs. Stress Pre)	0.047
Temp (Stress Pre vs. Stress Post)	0.039
ECG HR (Relax vs. Stress)	0.0
RSP RR (Relax vs. Stress)	0.008
∫EDA (Relax vs. Stress)	0.0
Slope EDA (Relax vs. Stress)	0.0
Lab results	
Cortisol (ΔRelax vs. ΔStress)	0.035
WBC (Stress Pre vs. Stress Post)	0.029
Psychological tests	
STAI-Y1 (Relax Pre vs. Relax Post)	0.0
STAI-Y1 (Stress Pre vs. Stress Post)	0.001
STAI-Y2 (ΔRelax vs. ΔStress)	0.002
ASI-3 (Relax Pre vs. Relax Post)	0.007

ECG, Electrocardiogram; EDA, Electrodermal activity; RSP, respiration; WBC, white blood cell.

### Correlations

**FIG. 13.**
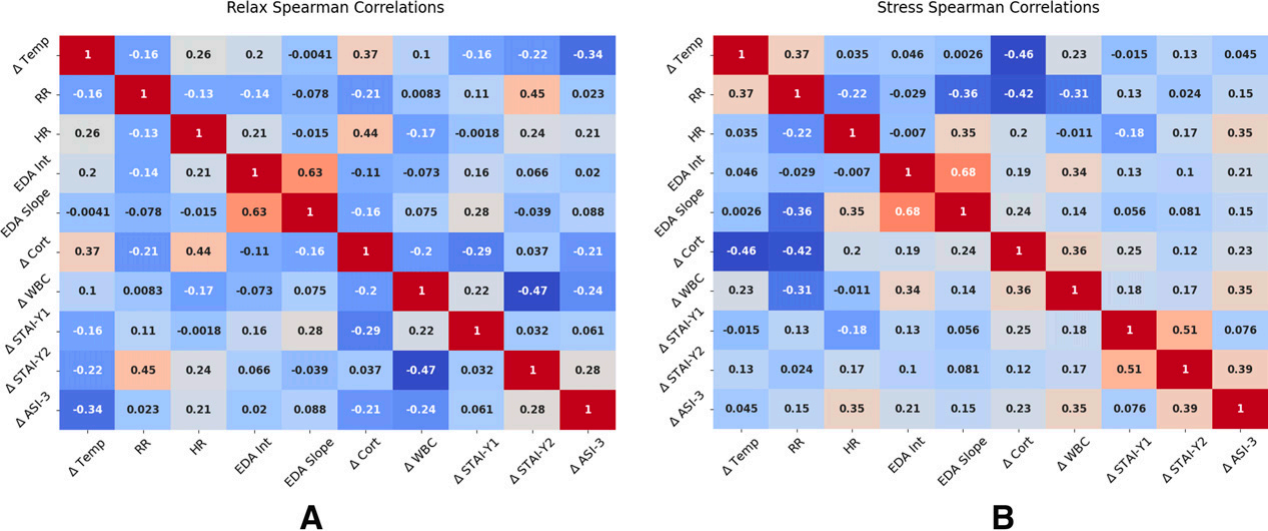
Spearman correlation results. The two heatmaps display the Spearman correlation values between all the physiological and psychological parameters for both experiences. For the parameters obtained before and after the experiences, we calculated the delta (post − pre). **(A)** Correlation results for the parameters obtained during the Relax VR experience. **(B)** Correlation results for the parameters obtained during the Stress VR experience. VR, virtual reality.

The correlation analysis for Relax VR showed a strong correlation between the EDA integral and EDA slope, which was an expected result. Additionally, we observed moderate to weak positive correlations between ΔTemperature and ΔCortisol, between RR and ΔSTAI-Y2, and between HR and ΔCortisol. We could also observe weak to moderate negative correlations between ΔTemperature and ΔASI-3, and ΔWBC and ΔSTAI-Y2 (see Fig. 13A).

Regarding the correlation analysis for Stress VR, similarly, we found a strong correlation between the EDA integral and the EDA slope. We also observed moderate to weak positive correlations between ΔTemperature and RR, HR and EDA Slope, HR and ΔASI-3, EDA integral and ΔWBC, ΔCortisol and ΔWBC, ΔWBC and ΔASI-3, ΔSTAI-Y1 and ΔSTAI-Y2, and ΔSTAI-Y2 and ΔASI-3. Additionally, there were some weak to moderate negative correlations between ΔTemperature and ΔCortisol, RR and EDA Slope, RR and ΔCortisol, RR and ΔWBC (see Fig. 13B).

## Discussion

The changes in our internal sensations resulting from exposure to stressful and relaxing VR experiences are manifested through changes in several physiological and psychological parameters. We explored the outcomes of exposing healthy volunteers to two different types of VR experiences (Relax and Stress) and assessed how they can induce or alleviate stress levels and their potential impact on the immune system.

Physiologically, exposure to the stressful VR experience led to an increase in skin temperature, as well as HR, RR, and sweat secretion. In line with previous reports,^33,34^ our results prove the effectiveness of VR to create vivid scenarios, in which the subjects feel deeply immersed leading to significant alterations of their physiological measurements depending on the nature of the experience. Moreover, the analysis of the body temperature pre-experience reveals disparities between the two experiences, potentially attributed to predispositions towards each type of experience. The psychological stress associated with anticipating participation in a stressful or relaxing experience may lead to changes in the human body temperature as indicated in previous studies.^35^

On the psychological levels, the self-assessment results confirm significant differences in the stress perception before and after both VR experiences. The STAI-Y1, which evaluates a person’s current or temporary anxiety levels, decreased in Relax VR and increased in Stress VR. This test proved crucial in assessing the volunteers’ stress perception during the two VR games. In Relax VR, the average pre-session score was 28, and decreased to 25 post-session, maintaining its values in the “low anxiety” range. Conversely, in Stress VR, the values increased from 30.65 to 40.45, shifting from a “low anxiety” level to a “moderate anxiety” level.

The STAI-Y2, measuring the individual’s general tendency to experience anxiety over time, exhibited statistical differences when comparing the delta (post − pre) for both experiences. In Relax VR, scores remained in the range of “low anxiety” pre- and post-experience (average pre: 36.85; average post: 33.25), while in Stress VR the values increased from the “low anxiety” levels (average pre: 35.5) to “moderate anxiety” levels (average post: 38.2). The individual’s tendency to experience anxiety overtime is increased by the exposure to a stress VR episode, whereas relaxing VR experiences may help maintain lower anxiety levels.

The final psychological test, ASI-3, demonstrated a significant difference for Relax VR but not for Stress VR. This test measuring the meaning of anxiety-relevant sensations helps identify what subscale between physical, social, and cognitive, generates the biggest concern for subjects. Across both experiences, the subscale consistently yielding the higher anxiety sensibility score was the social subscale, followed by physical and finally cognitive. Elevated social concerns typically relate to a fear of social rejection, likely due to the fear of negative evaluations.^36^ In Relax VR, scores for all three subscales notably decreased, whereas in Stress VR, the results remained unchanged. This underscores the potential efficacy of the relaxation meditation game in alleviating anxiety-inducing concerns.

Furthermore, our study explores the effect of the VR games in the blood cortisol levels. As an indicator of stress, cortisol levels showed a significant difference when comparing both experiences of net change (post − pre). In Relax VR, cortisol levels showed a slight average increase (0.8 mcg/dL). However, this increase was minimal compared to the substantial increase observed in the Stress VR session (2.55 mcg/dL). These results indicate an acute stress response of the human body to the stressful VR experience, marked by a steep increase in cortisol levels.

However, when comparing the four time points for the blood cortisol levels, these changes did not reach statistical significance. As expected from a heterogenous group of human subjects, we observed considerable variability in cortisol levels, likely explaining the lack of statistical significance in the results. This can at least partly be attributed to the adaptation to the VR system. While some subjects were more accustomed to the touch controls and adjusting to the headset, others experienced dizziness when using the technology. The sensation of dizziness was particularly pronounced during Stress VR, which involved a walk-through scenario. Despite having the ability to move around within the physical space, most subjects opted to stay stationary and relied on the joystick to navigate the virtual world. This dissociation of senses contributed to feelings of dizziness among participants. In this study, we did not track each individual’s VR experience level, which would have provided more information on how adaptation impacts physiological and psychological changes. This is an important consideration for future studies.

Research has shown that stress can significantly impact leukocyte levels in animals and humans. Therefore, we assessed the effects of Stress VR and Relax VR on leukocyte counts. In both experiences, the average WBC count increased from 6.6 × 10^3^/μL to 7 × 10^3^/μL. However, statistically significant differences were only observed when comparing pre- versus post-Stress VR. These changes were considerably less pronounced compared to those observed in mice, possibly due to the nature and duration of the acute stressor. Meanwhile, the Relax VR experience appears to alleviate stress as supported by the physiological data and the psychological test responses. However, a single session of Relax VR does not significantly impact the amount of WBC. To obtain more clinically significant changes, future studies could focus on repetitive VR sessions, potentially addressing chronic stress and mitigating adverse effects on immunity.

*In vivo*, individuals are frequently exposed to various stressors, leading to distinct physiological responses depending on the nature of the stressor. In this study, we examined the physiological effects of two VR experiences. Our findings revealed that the physiological response elicited by Stress VR is closely comparable to that of an acute *in vivo* stressor, indicating its potential to induce acute stress responses. Clinically, this could be leveraged in therapeutic contexts, such as for the treatment of phobias. Conversely, the Relax VR experience resulted in a significant reduction in RSP rate, HR, and sweat secretion. Psychological assessments further confirmed reductions in stress and anxiety levels, suggesting that Relax VR may be beneficial for managing chronic stress. Although a single session may not be sufficient to observe significant changes in the endocrine or immune systems, future research exploring repeated Relax VR sessions could demonstrate its potential to reconfigure the brain, ultimately alleviating unwanted symptoms related to stress.

We finally employed the Spearman correlation coefficient to examine the linear relationships among all physiological and psychological data for each type of experience. However, we did not find strong correlations between the parameters.

The study was not designed to directly benefit the participants but to unravel possible future benefits of VR experiences on the physiology and psychology of the users. This may lead, for example, to the development of VR “therapy” approaches to improve stress levels or the response to vaccinations.^37^ We can envision a future in medicine where comprehensive data tracking will coexist with the development of non-invasive regulatory systems designed to control and regulate pain or stress levels. While the mind possesses the power to influence our senses, it is crucial to recognize that we are in control of our minds, and VR stands as a potentially transformative tool to help reshape our emotional states.

This pilot study establishes the foundation for further exploring the development of VR therapy programs aimed at enhancing the adaptive immune response by supporting the regulation of an individual’s stress levels. We can envision potential applications involving the integration of this technology prior to vaccination programs, with the goal of boosting the efficacy of the vaccine. The active manipulation of stress levels at the time of vaccination, either by slightly increasing or reducing it, may improve the formation of a strong adaptive immune response.

## Limitations

We acknowledge that the sample size in this study poses a limitation for statistical analysis and for drawing definitive conclusions regarding the overall impact of these VR experiences on stress. Instead, the goal is to initiate exploration into stress responses in VR experiences, laying the groundwork for future, larger-scale research studies.

We also recognize that the game design of the selected VR games could have influenced the physiological parameters beyond the experience itself. The relaxing VR experience was conducted while seated and included breathing exercises which might have affected the respiratory results, although these exercises only constituted a small part of the whole relaxing experience. In contrast, the stressful VR experience was conducted standing up to enhance realism, but this might have also impacted physiological parameters.

The results presented should be considered within these limitations, and further research might be needed to validate the use of VR for stress treatment. For future research, it would be interesting to explore the effects of repeated VR sessions on stress levels management and its impact on the immune system. This strategy aligns with previous research that has demonstrated the efficacy of repeated VR interventions for pain management.^38^

## Data Availability

Anonymized psychological and physiological data would be available upon request.

## Abbreviations Used

ASI-3: Anxiety Sensitivity Index-3
ECG: Electrocardiogram
EDA: Electrodermal activity
FS: Flat screen
HR: Heart rate
PPG: photoplethysmography
RR: Respiratory rate
RSP: Respiration
SNS: Sympathetic nervous system
STAI: State-Trait Anxiety Inventory
VR: Virtual Reality
WBC: White blood cell
